# Rituximab Therapy for Refractory Autoimmune Thrombocytopenia in Patients with Systemic Lupus Erythematosus

**DOI:** 10.5505/tjh.2012.26539

**Published:** 2012-03-05

**Authors:** Didem Atay, Gülyüz Öztürk, Sema Anak, Ömer Devecioğlu, Ayşegül Ünüvar, Zeynep Karakaş, Leyla Ağaoğlu

**Affiliations:** 1 Okmeydani Education and Research Hospital, Department of Pediatric Hematology and Oncology, İstanbul, Turkey; 2 Istanbul University, Istanbul School of Medicine, Department of Pediatric Hematology, Oncology, BMT, İstanbul, Turkey

## TO THE EDITOR

Systemic lupus erythematosus (SLE) and autoimmunepolyendocrinopathy are characterized by autoantibodiesagainst a variety of target organs. Traditional treatmentstrategies for immune cytopenias include global immunosuppressionthat targets both the humoral and cell-mediatedarms of the immune system. B-cells play an importantrole in the pathogenesis of many autoimmune diseases.Targeted depletion of B-lymphocytes is an alternative treatmentapproach for autoimmune disorders. Rituximab—achimeric, human IgG1_K_ monoclonal antibody—is specificfor the CD20 antigen and can selectively deplete B-cellsvia antibody-dependent cell-mediated cytotoxicity, complement-mediated cytotoxicity, and inhibition of cell proliferationwith direct induction of B-cell apoptosis. Severalstudies have shown the efficacy and safety of rituximab inthe treatment of autoimmune disorders [1,2,3,4,5].

Herein we describe a 16-year-old male with SLE-associatedhemophagocytic syndrome (HPS), autoimmunehemolytic anemia (AIHA), and thrombocytopenia (AITP),as well as recurrent profound thrombocytopenia refractoryto glucocorticosteroids, intravenous immune globulin(IVIG) therapy, mycophenolate mofetil (MM F), and plasmapheresis.Four years after initially presenting with SLE,the patient was admitted to our hematology departmentwith severe thrombocytopenia and anemia; the hemoglobinlevel was 8.5 g dL^–1^, the white blood cell count (WBC)was 5000 mm^–3^, and the platelet (PLT) count was 3000mm^–3^. Poikilocytosis, anisocytosis, schistocytosis, spherocytosis,target cells, and few thrombocytes were observedin the blood smear. Lactate dehydrogenase was elevated(446 U L^–1^) and the reticulocyte count was 1.07%. Directantiglobulin test was 1(+) for complement (C3d) and IgG.C3 and C4 levels were decreased to 0,26 g/l and <0,10g/l. Antinuclear antibodies and anti-double stranded DNAantibodies were noted. Urinalysis showed urinary proteinexcretion of 2 g d^–1^.

Renal biopsy could not be performed because of thrombocytopenia.Bone marrow aspiration showed elevated histiocyteswith evidence of hemophagocytosis. The patientwas therefore diagnosed as SLE-associated HPS, AIHA,and AITP, and was treated with IVIG (0.4 g·kg^–1^·d–1 for5 d) and methylprednisolone (1 m g·kg^–1^·d^–1^), but severethrombocytopenia persisted (PLT count: 7000 mm^–3^).Pulse steroid (1 g d–1 dieb. alt.) and plasmapheresis (6times) were administered, but the patient did not respond(PLT count: 8000 mm–3). With MM F therapy (400 mgm–2 b.i.d.) the PLT count increased to 163,000 mm^–3^, butdecreased again to 1000 mm^–3^ after 3 months. Despite repeated high-dose steroid and IVIG treatment togetherwith MM F, a permanent response was not achieved.

Chronic corticosteroid treatment subsequently led togrowth retardation, osteoporosis, hypertension, glaucoma,and myopathy. Due to the side effects of corticosteroidsa slow tapering of oral steroids was started. Only twoweeks later, he relapsed again with severe thrombocytopenia.After written informed consent was obtained fromthe family, rituximab 375 mg m^–2^ QWK was started (intotal, 4 doses). Chlorpheniramine (0.02 mg kg^–1^ i.v.) andparacetamol (10 mg kg^–1^ p.o.) were administered 15 minprior to each rituximab infusion. This was well toleratedand the PLT count recovered to 240,000 mm^–3^ after 4weeks of the treatment (10,500 mm^–3^ after 1 week, 84,000mm–3 after 2 week, and 80,000 mm–3 after 3 weeks (Table1).

At the time this manuscript was written the patienthad been in remission for 49 months. The patient exhibitedslow regeneration of peripheral B-cells (total numberof CD19 and 20 cells)—although with subnormalcounts—49 months after treatment, and he was beingmaintained on a regimen of MM F and methylprednisolonefor lupus nephritis. The patient did not require immunoglobulinsubstitution due to rituximab therapy. To date,infectious complications have not been observed.

The efficiency of rituximab in patients with childhoodonsetSLE and severe autoimmune cytopenia has beenreported in a variety of series and case reports [6,7,8]. Thedrug was generally well tolerated; most children withAIHA or AITP remained disease-free. Treatment-relatedtoxicity is primarily due to infusion-related events. In thepresented case rituximab therapy was successful in treatingautoimmune cytopenias, but SLE remained active, aslupus nephritis and cutaneous lupus. As such, the persistenceof rituximab’s effectiveness in treating SLE remainsunclear. In conclusion, rituximab therapy shows promiseas an alternative to intensive immunosuppressive therapy and splenectomy in children with autoimmune cytopeniasthat are resistant to first-line therapy and cyclosporin/MM F. In cases of the recurrence of thrombocytopenia oranemia, a second treatment course is feasible and may successfullycontrol the disease. Additional research is necessaryto better understand the role of rituximab in thispatient population.

## CONFLICT OF INTEREST STATEMENT

The authors of this paper have no conflicts of interest,including specific financial interests, relationships, and/or affiliations relevant to the subject matter or materials included.

## Figures and Tables

**Table 1:**
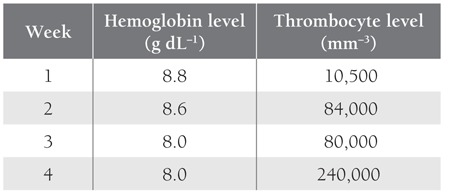
Hemoglobin and Thrombocyte Levels During RituximabTherapy
